# A salicylic acid derivative extends the lifespan of *Caenorhabditis elegans* by activating autophagy and the mitochondrial unfolded protein response

**DOI:** 10.1111/acel.12830

**Published:** 2018-09-07

**Authors:** Mehrnaz Shamalnasab, Simon‐Pierre Gravel, Julie St‐Pierre, Lionel Breton, Sibylle Jäger, Hugo Aguilaniu

**Affiliations:** ^1^ IGFL, UMR5262 Ecole Normale Supérieure de Lyon Lyon France; ^2^ Department of Biochemistry McGill University Montreal Quebec Canada; ^3^ Goodman Cancer Research Centre McGill University Montreal Quebec Canada; ^4^ Faculté de pharmacie Université de Montréal Montréal Quebec Canada; ^5^ Department of Biochemistry, Microbiology and Immunology, Faculty of Medicine University of Ottawa Ottawa Ontario Canada; ^6^ L’Oréal Research & Innovation Aulnay‐sous‐Bois France; ^7^ Instituto Serrapilheira Rio de Janeiro Brazil; ^8^ Détaché from CNRS (section 24) Paris France; ^9^ Present address: Warwick Integrative Synthetic Biology Centre (WISB), School of Life Sciences The University of Warwick Coventry UK

**Keywords:** autophagy, C. elegans, longevity, saliclic acid derivative, UPRmit

## Abstract

Plant extracts containing salicylates are probably the most ancient remedies to reduce fever and ease aches of all kind. Recently, it has been shown that salicylates activate adenosine monophosphate‐activated kinase (AMPK), which is now considered as a promising target to slow down aging and prevent age‐related diseases in humans. Beneficial effects of AMPK activation on lifespan have been discovered in the model organism *Caenorhabditis elegans *(*C. elegans*). Indeed, salicylic acid and acetylsalicylic acid extend lifespan in worms by activating AMPK and the forkhead transcription factor DAF‐16/FOXO. Here, we investigated whether another salicylic acid derivative 5‐octanoyl salicylic acid (C8‐SA), developed as a controlled skin exfoliating ingredient, had similar properties using *C. elegans* as a model. We show that C8‐SA increases lifespan of *C. elegans* and that a variety of pathways and genes are required for C8‐SA‐mediated lifespan extension. C8‐SA activates AMPK and inhibits TOR both in nematodes and in primary human keratinocytes. We also show that C8‐SA can induce both autophagy and the mitochondrial unfolded protein response (UPR^mit^) in nematodes. This induction of both processes is fully required for lifespan extension in the worm. In addition, we found that the activation of autophagy by C8‐SA fails to occur in worms with compromised UPR^mit^, suggesting a mechanistic link between these two processes. Mutants that are defective in the mitochondrial unfolded protein response exhibit constitutive high autophagy levels. Taken together, these data therefore suggest that C8‐SA positively impacts longevity in worms through induction of autophagy and the UPR^mit^.

## 1 INTRODUCTION

There are records dating back to thousands of years that mention the beneficial properties of extracts from willow barks. We now know that the main active molecules present in these trees are salicylates.

It is known that salicylates have beneficial activity on several pathways implicated in inflammation. For example, acetylsalicylic acid (ASA) is known to act as an anti‐inflammatory by inhibiting the activity of cyclooxygenase (COX) and thereby the production of prostaglandins (Wu, 2003). Interestingly, salicylates and other nonsteroidal anti‐inflammatory drugs were also shown to extend lifespan of yeast and fly through inhibition of tryptophan uptake (Danilov et al., 2015; He et al., 2014). Salicylates have also been shown to activate the adenosine monophosphate‐activated protein kinase (AMPK) (Hawley et al, 2012; Steinberg, Dandapani, & Hardie, 2013). This may in part explain why they held promise as treatments to improve insulin resistance and type 2 diabetes. AMPK has also been suggested to control the aging process in general (Salminen & Kaarniranta, 2012), and targeting AMPK has been discussed as a potential strategy to slow down aging in humans (Longo et al., 2015). Interestingly, ASA has recently been revealed as a lifespan‐extending treatment in both mice and nematodes (Ayyadevara et al., 2013; Strong et al., 2008; Wan, Zheng, Wu, & Luo, 2013). Salicylic acid also extends lifespan of *C. elegans,* albeit with a less pronounced effect than ASA (Ayyadevara et al., 2013). Work on the molecular mechanism in *C. elegans* has shown that activation of AAK‐2/AMPK and DAF‐16/FOXO was required for the lifespan‐extending activity of ASA (Wan et al., 2013). These results led us to investigate in the present work another salicylic acid derivate, 5‐octanoyl salicylic acid (referred to as C8‐SA), which was developed for its controlled skin exfoliating activity, as described in Saint‐Leger, Lévêque, & Verschoore, 2007. C8‐SA is a salicylic acid derivative containing an octanoyl group in meta‐position to the acid group (Supporting information Figure S1). Unlike for ASA or salicylic acid, no anti‐inflammatory activity has been detected for C8‐SA. However, we were able to show that C8‐SA displays a similar activity to ASA with regard to lifespan in the roundworm *Caenorhabditis elegans.*

## 2 RESULTS

### 2.1 C8‐SA extends Caenorhabditis elegans’ lifespan and health span by acting in somatic cells

**Figure 1:**
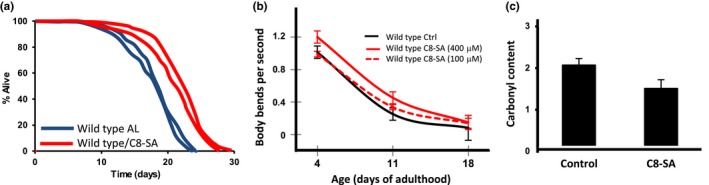
(a) Addition of 100 µM of C8‐SA extends the lifespan of wild‐type nematode *Caenorhabditis elegans* (N2). Two independent lifespan curves of each treatment are presented in the figure. (b) C8‐SA also improves the worms’ capacity to move at Day 11 and Day 18 of adulthood. In addition, this effect is more pronounced with 400 µM (*p* < 0.001) than with 100 µM (*p* < 0.001). (c) Concomitantly, the addition of the same amount of C8‐SA reduces significantly (*p* < 0.05) the overall level of carbonylated proteins

We first tested the impact of C8‐SA on lifespan using *C. elegans* as a model. This compound shows similar effects in *C. elegans* to other salicylic acid derivatives published earlier such as ASA and salicylic acid itself (Ayyadevara et al., 2013; Wan et al., 2013). Worms exposed to C8‐SA lived on average 19% longer than untreated controls (Figure 1a and Table 1) and remained healthier for longer periods of time. When we measured the number of body bends per second, we detected that treated animals moved more intensely than untreated animals especially at later stage in their lifespan (Figure 1b; Day 11 and Day 18 of adulthood). This tendency is statistically significant, and the positive effect of C8‐SA was dose‐dependent since 400 μM had a more pronounced effect than 100 μM (Figure 1b). Treating animals with 400 μM of C8‐SA did not lead to further enhancement of their lifespan (data not shown) In addition, treated animals showed reduced levels of carbonylated proteins, a hallmark of aging in *C. elegans *(Figure 1b). We observed that C8‐SA extended lifespan through its action on somatic cells. Indeed, we compared the effect of C8‐SA on the lifespan of wild‐type worms and of mutants carrying the *glp‐1(e2141ts) *allele that are defective in germline stem cell proliferation and grow to be fully germline less at 25°C (Arantes‐Oliveira, Apfeld, Dillin, & Kenyon, 2002). We found that C8‐SA extends the lifespan of the *glp‐1(e2141ts) *mutants to an extent similar to what we observed in wild type (Supporting information Figure S2 and Table 1). The finding that C8‐SA increases the lifespan of germline‐less worms is important because ablation of the germline has been shown to increase lifespan (Hsin & Kenyon, 1999).

**Table 1:** Lifespan data

Strain	Maximum lifespan	Mean lifespan ±*SEM* (days)	Number of worms (*N*)	*P*‐Value
WT	26	19.01 ± 0.28	543	
WT/C8‐SA	29	22.49 ± 0.33	511	<0.0001
WT	29	21.27 ± 0.47	120	
*daf* *−16* *(mu86)*	24	17.83 ± 0.25	166	<0.0001
*daf* *−16* *(mu86)*/C8‐SA	26	18.39 ± 0.25	167	<0.0001
WT	33	22.20 ± 0.40	120	
*isp* *−1* *(qm150)*	34	25.36 ± 0.34	140	<0.0001
*isp* *−1* *(qm150)*/C8‐SA	34	24.26 ± 0.26	175	0.0001
WT AL	31	20.52 ± 0.36	157	
WT BD (Bacterial deprivation)	44	31.73 ± 0.60	150	<0.0001
WT DR (Bacterial dilution)	38	24.06 ± 0.59	95	<0.0001
WT AL/C8‐SA	36	23.24 ± 0.38	144	
WT DR/C8‐SA	42	26.80 ± 0.62	133	<0.0001
WT BD/C8‐SA	36	22.23 ± 0.43	100	0.0994
WT	31	21.61 ± 0.45	123	
*bec* *−1* *(ok700)*	45	36.07 ± 0.51	149	<0.0001
*bec* *−1* *(ok700)*/C8‐SA	45	33.16 ± 0.71	145	<0.0001
WT	32	18.61 ± 0.38	124	
*hlh* *−30* *(tm1978)*	32	23.56 ± 0.41	113	<0.0001
*hlh* *−30* *(tm1978)*/C8‐SA	33	21.75 ± 0.56	104	<0.0001
WT	32	18.61 ± 0.38	124	
*hlh* *−30* *(OE)*	37	25.30 ± 0.62	115	<0.0001
*hlh* *−30* *(OE)*/C8‐SA	33	25.69 ± 0.59	119	<0.0001
WT	29	21.27 ± 0.47	120	
*ubl* *−5* *(ok3389)*	43	27.19 ± 0.72	140	<0.0001
*ubl* *−5* *(ok3389)*/C8‐SA	43	26.14 ± 0.73	137	<0.0001
*glp* *−1* *(e2141ts)*	52	33.81 ± 0.71	119	
*glp* *−1* *(e2141ts)*/C8‐SA	63	41.24 ± 0.89	119	<0.0001
*glp* *−1* *(e2141ts)*	43	29.09 ± 0.56	155	
*glp* *−1* *(e2141ts)*/C8‐SA	46	34.45 ± 0.51	119	<0.0001
WT	28	19.36 ± 0.35	119	
*aak* *−2* *(ok524)*	26	18.60 ± 0.33	112	0.0986
*aak* *−2* *(ok524)*/C8‐SA	29	18.67 ± 0.39	112	0.3209
WT	28	19.36 ± 0.35	119	
*aak* *−2* *(OE)*	28	22.86 ± 0.49	138	<0.0001
*aak* *−2* *(OE)*/C8‐SA	28	21.42 ± 0.56	125	0.0001

### 2.2 C8‐SA extends Caenorhabditis elegans’ lifespan through the insulin signaling pathway

**Figure 2:**
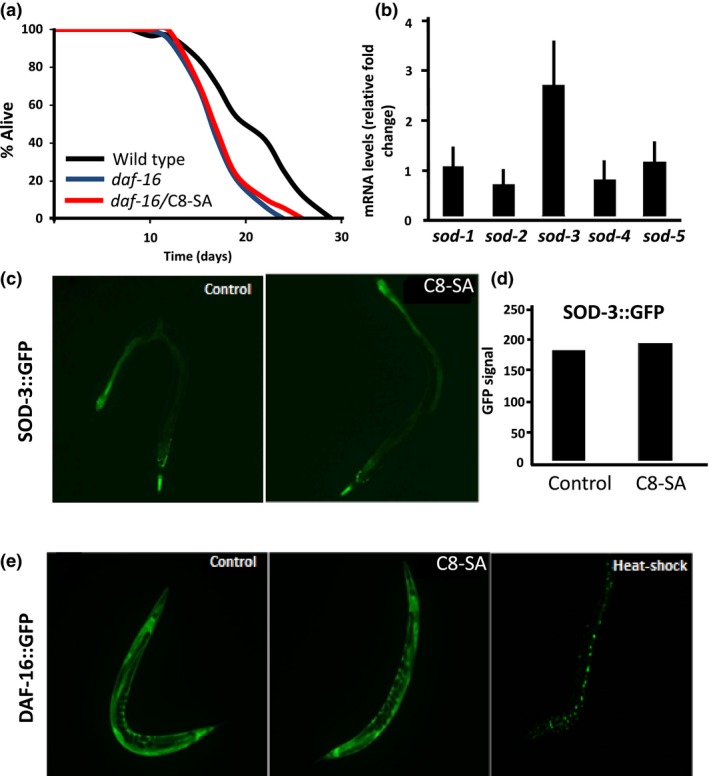
(a) C8‐SA fails to extend the lifespan of *daf‐16*/FOXO‐null mutants, suggesting that this molecule extends lifespan through daf‐16/FOXO. (b) In this context, upon C8‐SA treatment, the mRNA levels of the DAF‐16 target *sod‐3* are significantly induced (*p* < 0.01). (c and d) Surprisingly, SOD‐3::GFP levels are not increased under the same conditions, and (e) DAF‐16::GFP fails to translocate within intestinal nuclei (heat shock as positive control). The observed DAF‐16 transcriptional activation depends most likely on residual nuclear DAF‐16

We next asked whether any commonly known “aging pathways” were required for C8‐SA‐mediated lifespan extension. We first investigated the role of the insulin signaling pathway, which is known to regulate lifespan in multiple species (Kenyon, 2010). Similar to previous results using other salicylic acid derivatives (Ayyadevara et al., 2013; Wan et al., 2013), worms defective for the insulin signaling pathway, *daf‐16(mu86)* mutants, were unresponsive to C8‐SA. Indeed, *daf‐16(mu86)* mutants harbored similar mean and maximal lifespan when exposed to C8‐SA or the vehicle (Figure 2a and Table 1). We also found that C8‐SA treatment increased the endogenous mRNA levels of *sod‐3*, a DAF‐16/FOXO‐specific target (Figure 2b), even though SOD‐3 protein levels, when measured with the SOD‐3::GFP transgene, seemed to remain unchanged (Figure 2c,d). We also failed to detect the translocation of DAF‐16::GFP from the cytosol to nuclei of intestinal cells (Figure 2e). Although this may seem at odd with DAF‐16’s requirement for C8‐SA‐mediated lifespan extension and its action on *sod‐3* mRNA levels, it was already shown that DAF‐16 transcriptional activity could be boosted without altering DAF‐16::GFP localization (Xiao et al., 2013). Our data suggest that the presence of C8‐SA may activate DAF‐16‐dependent transcription without significantly altering the intracellular localization of the transgene.

### 2.3 C8‐SA extends Caenorhabditis elegans’ lifespan through the mitochondrial signaling pathway

Next, we asked whether C8‐SA extended lifespan through the mitochondrial pathway. It has been shown previously that altering the expression of some subunits of the mitochondrial complexes that mediate the oxidative phosphorylation within the inner membrane of the mitochondria could significantly extend the lifespan of *C. elegans *(Dillin et al., 2002; Lee et al., 2003). Interestingly, these interventions were then found to trigger the mitochondrial unfolded protein response (UPR^mit^) that contributes to the observed lifespan extension (Durieux, Wolff, & Dillin, 2011). Similar observations were also described in mice (Houtkooper et al., 2013; Jovaisaite, Mouchiroud, & Auwerx, 2014).

**Figure 3:**
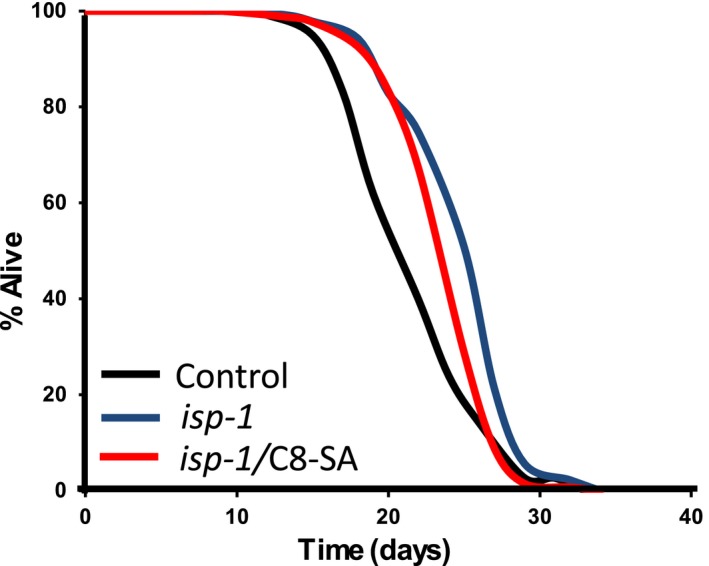
C8‐SA fails to extend the lifespan of *isp‐1* mutants, suggesting that the mitochondrial pathway is involved in C8‐SA‐mediated lifespan extension

We tested the impact of C8‐SA on worms mutated in *isp‐1* shown before to be long‐lived (Feng, Bussière, & Hekimi, 2001). The *isp‐1* gene encodes the Rieske iron‐sulfur protein of the mitochondrial respiratory chain complex III, and its mutation leads to reduced mitochondrial respiration (Feng et al., 2001). We found that, similar to *daf‐16(m86)*‐null mutants, C8‐SA failed to extend the lifespan of *isp‐1* mutants (Figure 3 and Table 1), suggesting that C8‐SA promotes lifespan at least in part through the iron‐sulfur protein *isp‐1*.

### 2.4 C8‐SA extends Caenorhabditis elegans’ lifespan through the dietary restriction pathway

**Figure 4:**
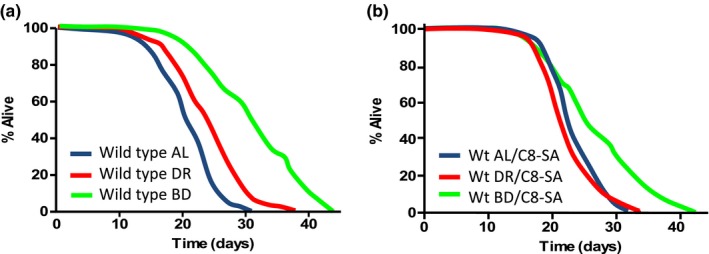
Treatment with C8‐SA strongly impairs the dietary restriction response. In this experiment, we used either bacterial deprivation (abbreviated BD; green curves) which is considered like a severe regimen of dietary restriction or bacterial dilution (abbreviated DR; red curves), considered as a mild dietary restriction. (a, b) It is striking that, in the presence of C8‐SA (b), the dietary restriction response is either partially or completely suppressed, depending on the severity of the regimen. This suggests that C8‐SA‐treated worms may be similar to wild‐type worms in dietary restriction

We also tested the impact of C8‐SA on the dietary restriction response. The restriction of food intake is one of the most powerful and robust ways to extend lifespan in a wide variety of organisms. Although this observation was first made by McCay in 1935 (McCay, Crowell, & Maynard, 1935), molecular mechanisms of nutritional‐mediated lifespan extension only started to be unraveled in 2007 (Bishop & Guarente, 2007; Panowski, Wolff, Aguilaniu, Durieux, & Dillin, 2007). We analyzed the lifespan of worms that were fed *ad libitum* or subjected to two different regimens of dietary restriction. In the first instance, we diluted the amount of bacteria (provided as a nutritional source for the nematodes) to which the nematodes were exposed. This intervention was already shown to extend lifespan (Greer et al., 2007). We also subjected worms to a complete bacterial deprivation as of their first day of adulthood. This experiment must be performed in the presence of fluorodeoxyuridine (FUDR) to inhibit matricidal hatching and results in an even more enhanced lifespan extension (Lee et al., 2006; Sutphin & Kaeberlein, 2008). In the presence of C8‐SA, bacterial dilution no longer extended lifespan, suggesting that lifespan is extended by C8‐SA and by dietary restriction through similar mechanisms (Figure 4a,b and Table 1). Thus, C8‐SA also acts in the dietary restriction pathway. More surprisingly, in extreme conditions of complete bacterial deprivation, C8‐SA decreased lifespan by 18%. At this stage, the causes of this apparent toxicity in such a challenging environment are unclear (Figure 4a,b and Table 1). In summary, C8‐SA seems to act on all longevity pathways known so far with the exception of the germline pathway.

### 2.5 C8‐SA activates various processes usually linked to dietary restriction

We next investigated the role of genes specifically known to be involved in the dietary restriction response. We first tested the impact of the forkhead transcription factor PHA‐4/FOXA known to be required for the dietary restriction response (Panowski et al., 2007). We found that *pha‐4* mRNA levels were induced upon dietary restriction in controls, but not in C8‐SA‐treated worms (Supporting information Figure S3a). In line with this, we also found that *pha‐4* targets were induced upon dietary restriction, but not in the presence of C8‐SA (Supporting information Figure S3b,c). Thus, C8‐SA treatment impairs the activation of the PHA‐4 pathway. This may explain, at least in part, why the lifespan extension observed upon DR is hampered when C8‐SA is administered to the worms.

We next confirmed that the AMPK pathway was also required for C8‐SA‐mediated lifespan extension, as it was shown for ASA (Wan et al., 2013). When C8‐SA was administered to mutants that either lacked or overexpressed the catalytic subunit of the AMPK, AAK‐2, lifespan remained unaffected (Supporting information Figure S4a,b and Table 1). The mean lifespan observed in these experiments was similar to that of wild‐type worms and therefore suggested that C8‐SA activates AMPK. To confirm that C8‐SA activates AMPK, we tested the impact of C8‐SA on the phosphorylation of its target ACC in primary normal human epidermal keratinocytes (pNHEK). We found that treatment with C8‐SA increased the level of pACC after 2 hr (Supporting information Figure S4c), suggesting that the C8‐SA effect on AMPK activity can be observed across species.

**Figure 5:**
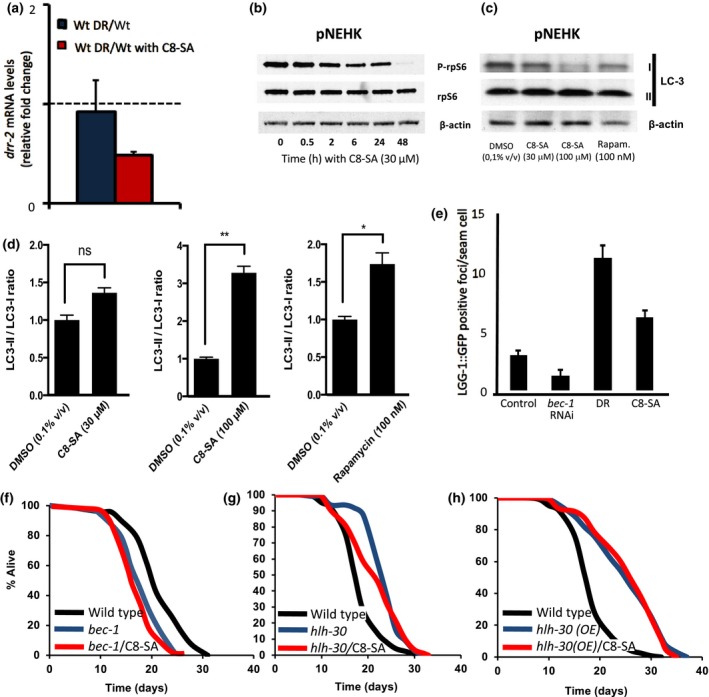
(a) C8‐SA inhibits the eIF4H *drr‐2* that acts downstream of TOR in worms and (b) reduces S6 phosphorylation in pNHEK and (c & d) induces autophagy as measured by the LC3‐II/LC3‐I ratio in pNHEK in a dose‐dependent manner. The induction of autophagy by 100 μM was actually found to be superior to that observed in response to exposure to 100 nM of rapamycin. In addition, C8‐SA (e) activates autophagy as detected by LGG‐1::GFP levels in hypodermal cells during the L3 stage. (f) Addition of C8‐SA failed to induce lifespan extension in worms in which *bec‐1*/beclin levels were downregulated. Similarly, the lifespan of worms in which the transcription factor *hlh‐30* was either downregulated (g) or upregulated (h) was insensitive to C8‐SA. Taken together, these results strongly suggest that C8‐SA alters lifespan by modulating autophagy levels

Since C8‐SA seems to act through most aging pathways and all of these pathways except the mitochondrial pathway implicate autophagy, we next investigated the action of C8‐SA on TOR signaling (that controls autophagy) and on autophagy itself. When we exposed the worms to C8‐SA, we could detect a significant reduction in the eukaryotic translation initiation factor 4H gene *drr‐2* that correlates with reduced TOR signaling (Figure 5a). This indicates that C8‐SA likely inhibits TOR signaling (Ching, Paal, Mehta, Zhong, & Hsu, 2010). Again, we tested the impact of C8‐SA in pNHEK on the phosphorylation state of the (ribosomal S6 protein), a key mTOR target. Accordingly, we also found that C8‐SA diminished the phosphorylation of the ribosomal S6 protein by mTOR (Figure 5b). Next, we found that C8‐SA induced autophagy in pNHEK by measuring LC3 I/II levels as well as in nematodes as measured by *lgg‐1/LC3*::GFP (Figure 5c–e). To test whether this induction was relevant to C8‐SA‐mediated lifespan increase in *C. elegans*, we then performed a lifespan experiment on worms treated with RNAi against the autophagy mediator beclin (*bec‐1*). We found that, under these conditions, C8‐SA no longer extended lifespan (Figure 5f). To further confirm these results, we next treated mutants that were either lacking or overexpressing the transcription factor *hlh‐30*. These worms are known to be impaired for autophagy or to display constitutively increased autophagy levels, respectively (Lapierre et al., 2013). In the two cases, C8‐SA lost its capacity to increase lifespan (Figure 5g,h). These results suggest that the positive impact of C8‐SA on lifespan and on health span may be transferable in human cells. Indeed, autophagy is induced both in worms and in human cells and is required for lifespan extension in worms. It will be interesting to gain further insights into the molecular mechanisms through which autophagy is activated by C8‐SA in future studies.

### 2.6 The UPR^mit^ is required for C8‐SA‐mediated lifespan extension

**Figure 6:**
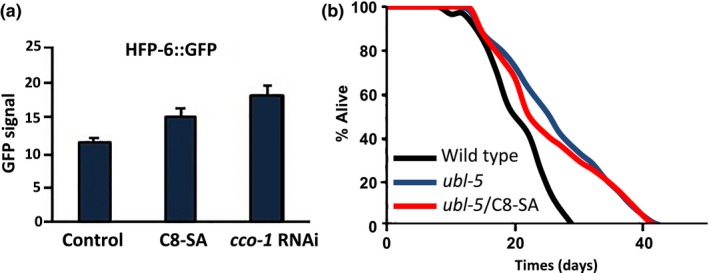
(a) C8‐SA activates the UPR^mit^ (*cco‐1* RNAi is used as a positive control) (b) Furthermore, C8‐SA fails to extend the lifespan of *ubl‐5* mutant worms that are defective for UPR^mit^. These results suggest that C8‐SA alters lifespan also by activating the UPR^mit^

As mentioned before, it has been shown that mutations in mitochondrial respiratory chain can trigger the mitochondrial unfolded protein response (UPR^mit^), which contributes to the lifespan extension observed (Durieux et al., 2011). Because C8‐SA also acts through the mitochondrial pathway (Figure 3), similar to ASA (Wan et al., 2013), we next set out to test the action of C8‐SA on the UPR^mit^. We could detect a small increase in the UPR^mit^ readout, HSP‐6::GFP, even though not statistically significant (Figure 6a). To better test the role of the UPR^mit^ on C8‐SA‐mediated lifespan extension, the impact of C8‐SA on *ubl‐5* mutants that are defective in the UPR^mit ^(Durieux et al., 2011) was evaluated. We found that C8‐SA was unable to extend the lifespan of *ubl‐5* mutant worms (Figure 6b and Table 1), suggesting that the UPR^mit^ plays an important role in C8‐SA‐mediated lifespan extension.

### 2.7 Autophagy and UPR^mit^ are linked

**Figure 7:**
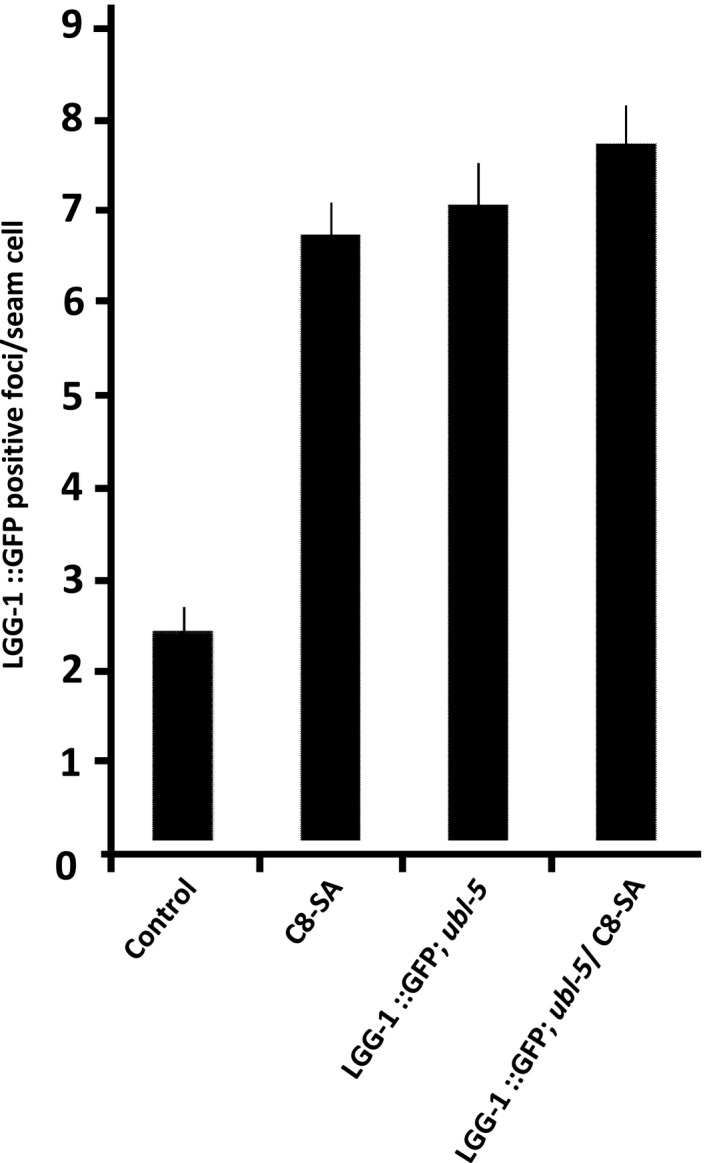
C8‐SA activates autophagy in wild‐type worms, but fails to increase autophagy in worms that are defective for the UPR^mit^ (*ubl‐5* mutants). Interestingly, the sole genetic ablation of ubl‐5 sufficed to induce autophagy monitored by increased LGG‐1::GFP levels. This reveals an unforeseen interaction between autophagy and the UPR^mit^

Finally, we wondered whether the respective activation of Auto‐phagy and the UPR^mit^ was somewhat linked. Although some stresses such as dietary restriction are known to trigger the UPR^mit^ and autophagy in parallel in worms (Bennett et al., 2017) and the UPR^mit^ was recently linked to DR and its downstream effectors in Drosophila (Borch Jensen, Qi, Riley, Rabkina, & Jasper, 2017), the impact of UPR^mit^ on autophagy independently of stress has never been described. In order to address this question, we generated an *ubl‐5 *mutant expressing LGG‐1::GFP to monitor autophagy levels *in vivo*. We found that the *ubl‐5* mutation harbored significantly higher autophagy levels than control worms (Figure 7). In addition, C8‐SA did not further increase autophagy levels in these UPR^mit^‐defective mutants (Figure 7). C8‐SA therefore activates both UPR^mit^ and autophagy in wild‐type animals, and both of these processes are required for C8‐SA‐mediated lifespan extension.

Taken together, our data suggest that autophagy is either inhibited by the UPR^mit^ or that, in the absence of a functional UPR^mit^, autophagy is constitutively activated in the worm. In addition, C8‐SA fails to further activate autophagy in the absence of UPR^mit^. Thus, C8‐SA requires the presence of both autophagy and UPR^mit^ in order to affect lifespan in a positive way; however, these requirements do not seem to be independent of one another. It is particularly intriguing to observe that, in C8‐SA‐treated *ubl‐5* mutants, autophagy levels are not different from that of C8‐SA‐treated wild‐type animals (Figure 7). Our data therefore suggest that activating autophagy is insufficient for C8‐SA to increase lifespan. Instead, C8‐SA requires a functional UPR^mit^ to be effective even if this implies lower autophagy basal levels.

## 3 DISCUSSION

In this work, we investigated the impact of 5‐octanoyl salicylic acid on the health and the lifespan of the nematode *Caenorhabditis elegans*. It is important to note that there have been earlier reports showing that both acetylsalicylic and salicylic acids could extend *C. elegans*’ lifespan (Ayyadevara et al., 2013; Wan et al., 2013). In these reports, several genes were shown to be important for the lifespan effect of these compounds, including the AMPK, the forkhead transcription factor DAF‐16/FOXO and other genes involved in the oxidative stress response. Here, using yet another salicylic acid derivative (C8‐SA), we confirm that these molecules do extend lifespan in a remarkable way. Indeed, we show that C8‐SA extends lifespan solely acting on somatic tissues since its effect is still observed in worms lacking a germline. We also found that most aging pathways were required for C8‐SA‐mediated lifespan extension. Mutants defective for *daf‐16* were incapable to respond to C8‐SA, although C8‐SA failed to induce DAF‐16 translocation from the cytosol to the nuclei. We also found that *isp‐1* and *aak‐2*/AMPK mutants were unresponsive to C8‐SA. Taken together, these observations are puzzling as they indicate that C8‐SA acts through many of the known longevity genes.

We also detected that C8‐SA exposure led to the activation of AMPK and to the inhibition of TOR signaling in worms and in primary human keratinocytes. This led us to investigate the role of C8‐SA on the induction of autophagy and to confirm that C8‐SA significantly activates autophagy both in worms and in primary human keratinocytes. Also, when treated with *bec‐1 *RNAi that disrupts autophagy, C8‐SA becomes less effective, suggesting that its effect on lifespan requires this process. Although it is possible that more specific processes such as mitophagy are activated by C8‐SA, our experiments do not allow to discriminate between autophagy and mitophagy. It has been described before that autophagy plays an important role in the keratinization process during epidermal differentiation (for review see Li, Chen, & Gu, 2016). It is therefore possible that C8‐SA might have antiaging benefits on the skin beyond its controlled exfoliating activity.

The requirement of the *isp‐1* for C8‐SA‐mediated lifespan extension also led us to investigate the role of the UPR^mit^. Although, the UPR^mit^ readout HSP‐6::GFP only exhibited a small induction upon C8‐SA treatment, we found that C8‐SA was unable to enhance the lifespan of *ubl‐5 *mutants that are UPR^mit^‐defective. Thus, both autophagy and UPR^mit^ are required processes for C8‐SA‐mediated lifespan extension.

We finally asked whether these processes were linked or independent. To this end, we constructed transgenic worms defective in the mitochondrial unfolded protein response (*ubl‐5* mutants) carrying an autophagy reporter (*lgg‐1*::GFP). Thanks to these, we found that the ablation of the UPR^mit^ led to a constitutive increase in autophagy. One explanation for this observation is that autophagy may take over when UPR^mit^ is abrogated to compensate the loss of its machinery. Interestingly, when these nematodes were treated with C8‐SA, autophagy levels remained unaffected. These data suggest that the induction of autophagy by C8‐SA requires the presence of an integer UPR^mit^ and therefore advocates in favor of an intrinsic link between the autophagy and the UPR^mit^ processes. To our knowledge, the data presented here provide the first evidence for the existence of such a link.

## 4 MATERIAL AND METHODS

### 4.1 Nematode maintenance and strains

Strains were cultured under standard laboratory conditions. The wild‐type N2 (Bristol) was used as the reference strain. *C. elegans* strains are listed below (name, genotype, and origin):

AD105, *daf‐16(mu86) I,* CGC *
TJ356, *zIs356 IV [daf‐16::GFP* *+pRF6* *], *CGC
DA2123, *adIs2122 *[*lgg‐1::GFP* *+pRF6* *]*, Hansen Lab
CF1903, *glp‐1(e2141ts)* III, Kenyon Lab
VC2654, *ubl‐5(ok3389)* I, CGC
SJ4100, zcIs13[*hsp‐6*p∷GFP],CGC
MQ887, *isp‐1(qm150)* IV, CGC
CF1553, muIs84 [(pAD76) sod‐3p::GFP +rol‐6(su1006)], CGC
RB754, *aak‐2(ok524) *X, CGC
WBM60, *uthIs248 [aak‐2p::aak‐2(genomic aa1* *–* *321)::GFP::unc‐54 3'UTR* *+myo* *‐2p::tdTOMATO], *Mair Lab
JIN1375, *hlh‐30(tm1978) IV, outcrossed six times to irazoqui Lab (JIN) N2*, Hansen Lab
MAH235, *sqIs19[hlh‐30p::hlh‐30::gfp, rol‐6(su1006)],* Hansen Lab
VC424, *bec‐1(ok700) IV/nT1 [qIs51] (IV;V),* CGC
*ubl‐5(ok3389)* I; *zIs356 IV [daf‐16::GFP* *+pRF6* *], *made in the laboratory
  *CGC = Caenorhabditis Genetics Center

### 4.2 Lifespan analysis

Lifespan assays were conducted at 20°C according to standard protocols. After bleaching, aged synchronized eggs were grown in M9 buffer overnight at 20°C and put on plates at the L1 stage. Worms were maintained on solid nematode growth medium (NGM) containing 25 µg/ml carbenicillin and 15 µM 5‐fluorouracil and seeded with *Escherichia coli* strain HT115. At Day 1 of adulthood, 120 worms were transferred to plates seeded with dead *Escherichia coli *and treated with ethanol or C8‐SA (100 µM) in ethanol. Worms that failed to display heat‐provoked movement were scored as dead. Statistical analysis and mean lifespan were obtained by Oasis software. P‐values were calculated using a log‐rank test.

### 4.3 *Caenorhabditis elegans* movement assay

Wild‐type N2 *C. elegans* worms were grown on Nematode Growth Medium (NGM Agar) prepared in 24‐well plates (1.5 ml per well). Treatment compounds were prepared as solutions in DMSO, of which 10 µl was added to each well to achieve the final desired treatment concentration in the media. Once the treatments had dried, each well was seeded with OP50 *E. coli* culture, and plates were incubated at 20°C for 24 hr before approximately 15 *C. elegans* eggs were added to each well. Plates were maintained in these conditions for approximately three days until the larvae reached the L4 development stage, at which point the plates were treated with 133 µM FUDR. Plates were then maintained at 20°C with sufficient food (*Escherichia coli* strain OP‐50) until imaging.

Eight individual wells were imaged per treatment at each of the respective timepoints (Days 4, 7, and 11 of adulthood). For the imaging, any excess worms were first removed to leave 10 per well, and wells were then flooded with 200 µl of M9 buffer. Worms were allowed 30 s to adapt, before 30‐s videos were captured at 30 frames per second with StreamPix 6 software (Norpix), using an Infinity 2 CCD camera (Luminera) on top of a Motic SMZ‐171 microscope. Images were then analyzed using ImageJ according to the *C. elegans* motility analysis protocol (Pedersen, 2017). The data were filtered to remove any worms for which less than 15 s of movement data was available before further statistical analysis was completed.

Three replicate experiments were completed, and the data were analyzed in R (version 3.4.1). A fixed‐effects model was fitted to the data to estimate the effect of treatments on the number of BBPS using the lme4 package, with replicate experiment included as a random effect, and Tukey posttests completed to allow specific comparisons between treatments using the emmeans package.

### 4.4 Dietary restriction

DR was performed through bacterial deprivation or bacterial dilution from the first day of adulthood.

### 4.5 Bleaching method

Worms were suspended in 5 ml M9 buffer and washed once. After adding 200 µl 5 N NaOH and 500 µl bleaching solution, the worms were vortexed for 6 min and centrifuged for 5 min at maximum speed (400g) to explode adult worms. M9 buffer was then added up to 10 ml, centrifuged at maximum speed for 2 min at +4°C, and the pellet was washed four times with 10 ml M9 buffer. Eggs were then transferred to a 250‐ml flask with 50 ml M9 buffer and incubated at 20°C overnight.

### 4.6 qRT–PCR

For each gene, analyses were performed on triplicate biological samples and for each sample, two technical replicates.

### 4.7 RNA extraction and purification

Total RNA was isolated from synchronized populations of Day 1 adult worms (about 3,000 individuals per condition) using the following method. Worms were harvested and washed three times with M9 buffer and twice with DEPC water. TRIzol reagent (MRC) was added to the worm pellet (TRIzol/worm pellet ratio was 2/1), and the mixture was vigorously shaken for at least 1 min. The mixture was frozen at −80°C overnight or for a longer period before the next RNA extraction steps.

Frozen worms were then placed on ice, vortexed for 5 min, and settled at room temperature. The TRIzol/worm mix was transferred to Eppendorf tubes, and chloroform (200 µl per 1 ml TRIZOL/worm mix) was added to each tube. Tubes were shaken for 15 s, incubated at room temperature for 2 min, and centrifuged at 12,000 *g* for 5 min. The upper aqueous phase was transferred to a new tube. An equal volume of 70% ethanol (prepared in DEPC water) was added to the aqueous phase, and tubes were gently mixed by inversion.

RNA extraction was then performed following the RNeasy (Qiagen, Courtaboeuf, France) kit instructions, including the optional step of DNase digestion on the column. RNAs were eluted in 50 µl RNase‐free water. RNA concentrations were determined using a NanoDrop spectrophotometer. RNA extracts were used directly or kept at −80°C.

### 4.8 RNA reverse transcription

cDNAs were created using the iScript cDNA Synthesis Kit (Bio‐Rad) using 500 ng of RNA.

### 4.9 Quantitative real‐time PCR

SYBR Green (Bio‐Rad, Marnes‐la‐Coquette, France) real‐time qPCR experiments were performed as described in the Step One Plus manual. 10 ng of cDNA was used for qPCR, the equivalent of 2 µl of cDNA in each well. 14 µl of Master Mix (8 µl of SYBR Green, 4.4 µl of DEPC water, and 1.6 µl of primer pair) was added in each well. Quantitative qRT–PCR reactions were carried out on a Light Cycler 1.5 (Roche, Mannheim, Germany). The standard curve method was used to determine the relationship between mRNA abundance and PCR cycle number.

### 4.10 DAF‐16 localization assays

On Day 1 of adulthood, worms carrying a *daf‐16*::GFP transgene were assayed for DAF‐16 nuclear localization in intestinal cells using a Nikon Eclipse 80i fluorescent microscope (Europe B.V., Badhoevedorp, the Netherlands) at 400x magnification. For the heat‐stress challenge, worms were shifted to 37°C for 1 hr. Worms were scored as having nuclear DAF‐16 if the majority of intestinal cells displayed a distinct concentration of GFP in the nucleus. Approximately 15–30 worms were analyzed for each condition.

### 4.11 Analysis of autophagy levels using an LGG‐1 reporter strain

Autophagy was monitored using an *lgg‐1*::GFP translational reporter. GFP‐positive puncta in seam cells was counted in L4 transgenic worms using a Leica DMI 6,000 B microscope (Leica, Nanterre, France) at 1,000× magnification. All worms were kept at 20°C and were treated with ethanol (as a control), and C8‐SA and then their progeny were assayed at the L3 stage. The number of puncta per seam cell was averaged for each worm, and this average was used for calculating the population mean number of LGG‐1::GFP‐containing puncta per seam cell.

### 4.12 Immunoblot analyses of primary human keratinocytes extracts

pNHEK (C‐12003, PromoCell, Heidelberg, Germany) were maintained in keratinocyte growth media (C‐20111, PromoCell). Subconfluent cells (Passage 4 to 8) were treated with DMSO 0.1% (v/v), C8‐SA at 30 µM/100 µM, or rapamycin (100 nM). At endpoint, cells were washed with cold phosphate buffer saline 1X and lysed in RIPA lysis buffer (Tris–HCl 50 mM, NaCl 150 mM, EDTA 5 mM, Triton 1%, glycerol 10%, pH 7.4) supplemented with sodium orthovanadate 1 mM, sodium fluoride 50 mM, β‐glycerophosphate 40 mM, pepstatin 1 µg/ml (Roche, Laval, Canada), aprotinin 2 µg/ml (Roche), leupeptin 1 µg/ml (Roche), and Pefebloc 1 µg/ml (Roche). Lysates were cleared by centrifugation, and protein concentration was assessed using the Pierce BCA protein assay (Thermo Scientific, Waltham, MA, USA). 30 µg of total protein extracts was loaded on 4%–12% Criterion XT Bis‐Tris precast gels (Bio‐Rad, Mississauga, Canada). Proteins were immobilized on PVDF membranes, which were blocked in 5% nonfat dry milk in Tris‐buffered saline with Tween‐20 0.1% (v/v) prior to immunoblotting. The following antibodies were used for immunoblotting: Cell Signaling Technology (Danvers, MA, USA): phospho‐ACC (#3,661), ACC (#3,662), phospho‐S6 ribosomal protein Ser235/236 (#2,211), and S6 (#2,271), LC3‐A/B (#4,108); Santa Cruz Biotechnology Inc. (Dallas, TX, USA): β‐actin (sc‐1616). HRP conjugates (Mandel, Guelph, Canada) were detected using the Western Lightning Plus‐ECL detection system (PerkinElmer, Waltham, MA, USA).

### 4.13 GFP expression and quantification

SOD‐3::GFP activity was assayed using strain CF1553 (*muIs84*). HSP‐6::GFP activity was assayed using strain SJ4100 (zcIs13). Age‐synchronized (Day 1 of adulthood), transgenic worms were treated with ethanol as the solvent control and C8‐SA (100 µM), for 24 hr. Total GFP signal for each worm was quantified by ImageJ software. Data shown are the average number of pixels in the transgenic *C. elegans* (*n* = 10–15) at each indicated treatment. Data are presented as the mean ± *SEM*.

### 4.14 Protein carbonylation

Protein extracts were prepared from 1‐mL pellet of synchronized Day 2 adult nematodes grown on ht115 plates. All protein samples were resolved by electrophoresis through 10% gradient SDS‐polyacrylamide gel. Proteins were detected by immunoblot using the OxyBlot Protein Oxidation Detection Kit following the provided protocol (Aguilaniu, Gustafsson, Rigoulet, & Nyström, 2003). Detections were accomplished using the ECL Plus Western Blotting Detection System following the provided instructions. Band intensities were quantified with ImageJ. OxyBlot analyses were repeated twice with lysates from separate nematode preparations.
