# Corrigendum

**DOI:** 10.1111/acel.12917

**Published:** 2019-03-12

Shamalnasab M, Gravel S‐P, St‐Pierre J, Breton L, Jäger S, Aguilaniu H. A salicylic acid derivative extends the lifespan of Caenorhabditis elegans by activating autophagy and the mitochondrial unfolded protein response. Aging Cell. 2018;17:e12830. https://doi.org/10.1111/acel.12830

In the article “A salicylic acid derivative extends the lifespan of Caenorhabditis elegans by activating autophagy and the mitochondrial unfolded protein response”, the authors would like to correct the value of maximum and mean lifespan “bec−1(ok700)” and “bec−1(ok700)/C8‐SA” in Table 1 from “45” to “25” for maximum lifespan and 19.17±0.51 and 18.06±0.51 for mean lifespan, respectively.

The authors would like to apologize for the inconvenience caused.

