# Supplementary material for: A salicylic acid derivative extends the lifespan of Caenorhabditis elegans by activating autophagy and the mitochondrial unfolded protein response
Source: Aging Cell. 2018 Sep 7;17(6):e12830. doi: 10.1111/acel.12830 (PMC6260907; doi:10.1111/acel.12830)

Supplementary Figure 1

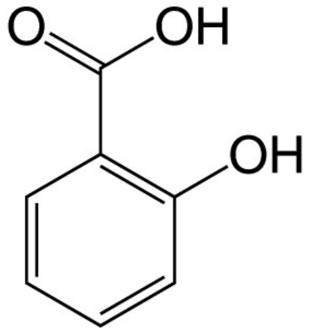

Salicylic acid

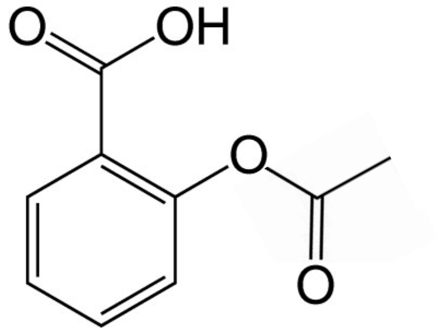

Acetylsalicylic acid

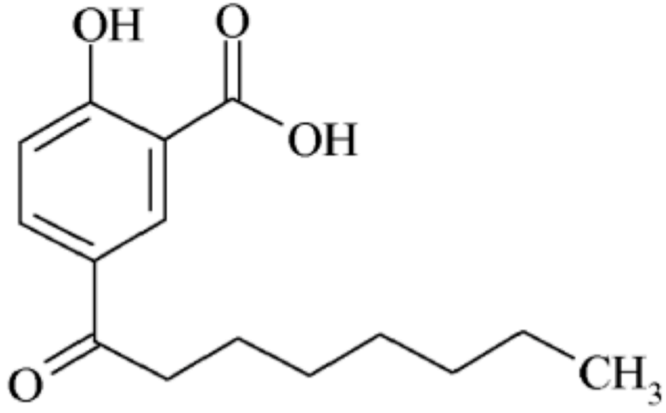

5-octanoyl salicylic acid

Supplementary Figure 2

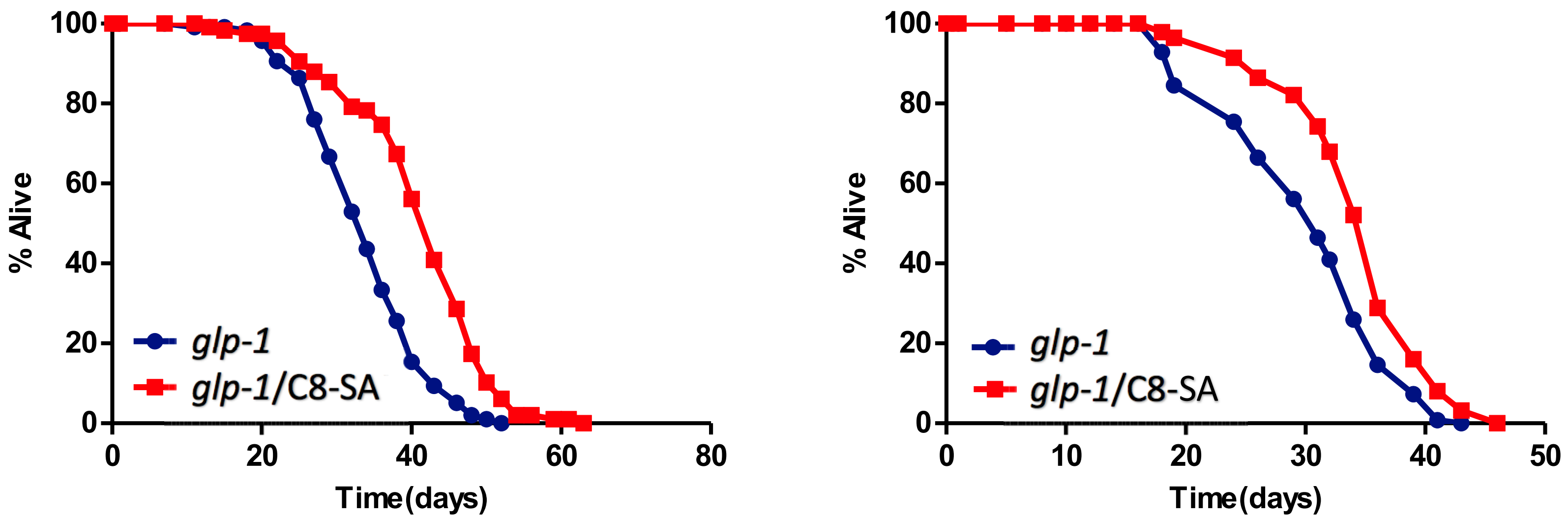

Supplementary Figure 3

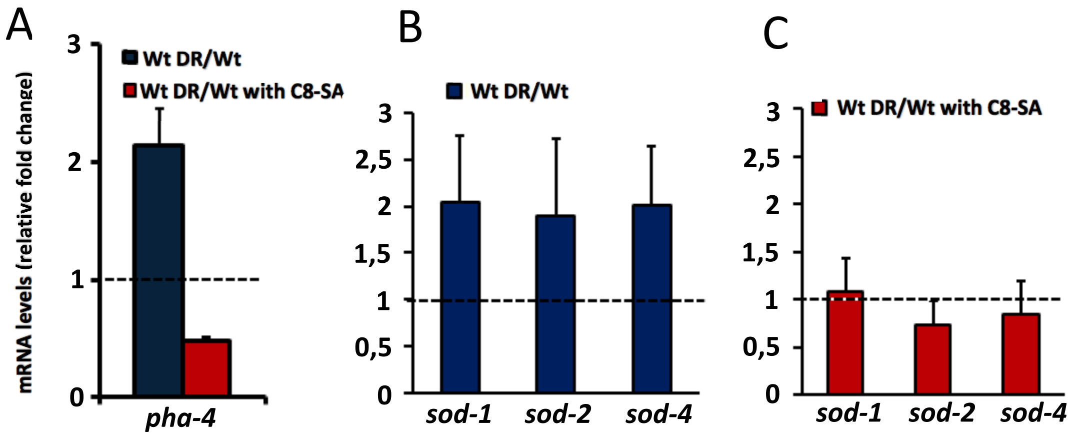

Supplementary Figure 4

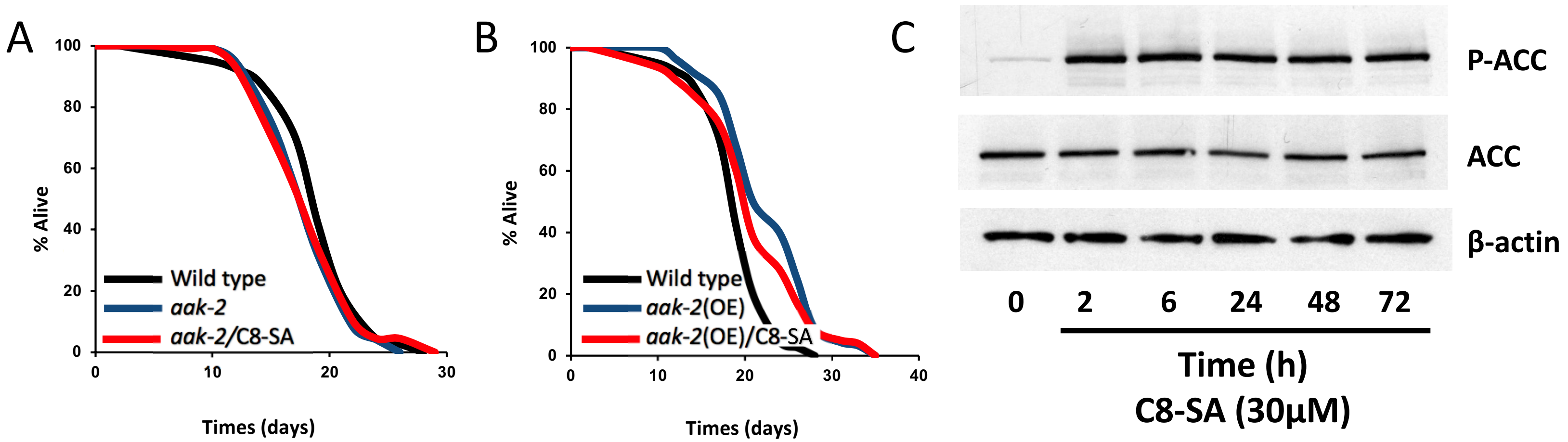

Supplement: Supplementary file 1 [file ACEL-17-e12830-s001.pdf]
